# Click Chemistry: Reaction Rates and Their Suitability for Biomedical Applications

**DOI:** 10.1021/acs.bioconjchem.4c00084

**Published:** 2024-05-22

**Authors:** Tracey Luu, Katie Gristwood, James C. Knight, Manuela Jörg

**Affiliations:** † Medicinal Chemistry Theme, Monash Institute of Pharmaceutical Sciences, Monash University, Parkville, Victoria 3052, Australia; ‡ School of Natural & Environmental Sciences, Newcastle University, Newcastle Upon Tyne NE1 7RU, U.K.

## Abstract

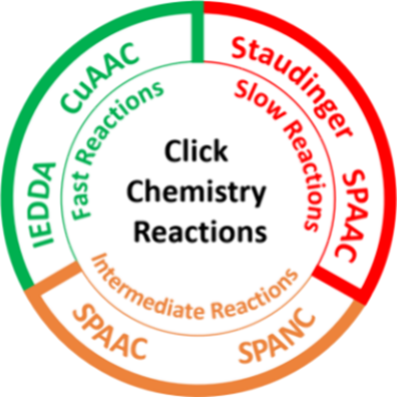

Click chemistry has become a commonly used synthetic method due to the simplicity, efficiency, and high selectivity of this class of chemical reactions. Since their initial discovery, further click chemistry methods have been identified and added to the toolbox of click chemistry reactions for biomedical applications. However, selecting the most suitable reaction for a specific application is often challenging, as multiple factors must be considered, including selectivity, reactivity, biocompatibility, and stability. Thus, this review provides an overview of the benefits and limitations of well-established click chemistry reactions with a particular focus on the importance of considering reaction rates, an often overlooked criterion with little available guidance. The importance of understanding each click chemistry reaction beyond simply the reaction speed is discussed comprehensively with reference to recent biomedical research which utilized click chemistry. This review aims to provide a practical resource for researchers to guide the selection of click chemistry classes for different biomedical applications.

## Introduction

**Figure 1:**
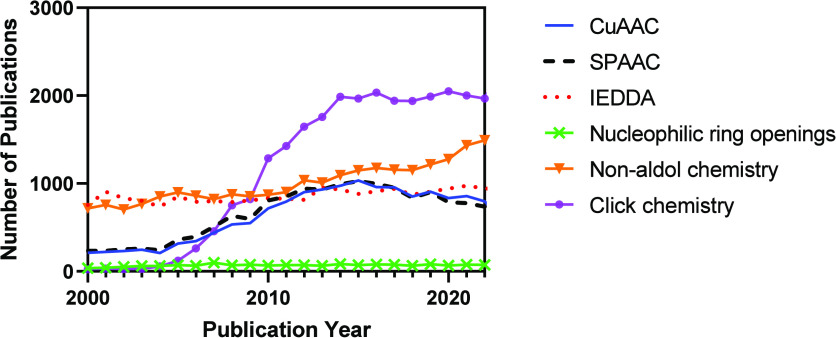
Number of publications containing the key word “CuAAC”, “SPAAC”, “IEDDA”, “nucleophilic ring opening”, “non-aldol carbonyl reactions”, and “carbon–carbon multiple bond additions” from 2000 to 2023.^7^

Click chemistry, a term initially coined by Sharpless and colleagues,^1^ involves a selection of reactions that produce simple, fast, and high-yield compounds. Other criteria include the modularity, wide applicability, and the formation of benign byproducts. In the past two decades, click chemistry has shown to be an appealing and reliable strategy (Figure 1) that has provided an alternative synthetic approach to existing chemistry methodologies. Conventionally, the preparation of novel materials required labor-intensive, multistep synthetic procedures, which were often hindered by non-specific side reactions involving competing functional groups.^2^ Click chemistry has emerged as a strategic response to overcome these challenges, providing an efficient method for the synthesis of a wide array of molecules. Hence, click chemistry has found applications in various chemistry disciplines, including radiochemistry,^3^ polymer chemistry,^4^ peptide chemistry,^5^ and pharmaceutical chemistry.^6^

In recent years, click chemistry has found applications in the study of biological systems. Hence, the term bioorthogonal chemistry was coined.^8^ In addition to the criteria of click chemistry, bioorthogonal chemistry requires the reactions to form nontoxic products that are stable in vivo and do not interfere with endogenous biological processes.^9^ Despite the progress in the field, there are still a limited number of in vivo applications, including reports of antibody–drug conjugates,^10^ fluorescence imaging,^11^ and drug delivery for targeted cancer therapies.^12^ One of the challenges in developing novel biomedical applications is the selection of the bioorthogonal chemistry method that is most appropriate for the intended application. A number of factors need to be taken into consideration, including selectivity, reactivity, biocompatibility, and stability. An important aspect that is often overlooked is the influence of the rate of reactions. A common misconception is that high reaction rates are necessary for any biomedical application and to maximize clinical impact of an application.^13^ However, this assumption does not always hold true. The assessment and understanding of click chemistry reactions should extend beyond the need for fast rates; instead, it should be tailored to the specific applications and research aim.

The importance of click chemistry reaction rates for drug delivery applications have recently being discussed and analyzed by Kondengadan et al.^14^ In this complementary review, we provide the novice researcher in different fields of biomedical sciences, including medicinal chemistry, pharmacology, and biochemistry, with an introduction to click and bioorthogonal chemistry. A brief overview of well-established, less conventional, and newer click chemistry reactions highlights the advantages and limitations of these different classes of click chemistry reactions. Furthermore, the review compares reaction rates of different click chemistry classes and evaluates the suitability of reactions for biomedical applications. Finally, examples of recent studies that highlight the impact of reaction rates have been included for a broad range of biological and biomedical applications. Overall, the review has a particular focus on the importance of reaction rates and guides nonexperts to make an informed decision on which click reaction will be optimal for their applications.

## Click Chemistry Reaction Classes

This section summarizes the key characteristics of click chemistry reactions, serving as a practical resource for researchers to select the most suitable reaction for their specific biomedical applications, including radiolabeling, formation of biopolymer materials, drug delivery systems, and drug development more broadly. The four most established types of click chemistry reactions include: cycloadditions, nucleophilic ring-opening reactions, non-aldol carbonyl reactions, and additions to carbon–carbon multiple bonds.^15,16^

### Cycloadditions

Cycloadditions are primarily 1,3-dipolar and hetero-Diels–Alder reactions and are most commonly used in bioorthogonal chemistry (Table 1). Copper(I)-catalyzed azide–alkyne click chemistry (CuAAC), the first-generation cycloaddition, utilizes a copper(I) catalyst to facilitate a reaction between an azide and an alkyne (Table 1, entry 1). These reactions demonstrate the second-highest rate constant, ranging between 10 to 10^4^ M^–1^ s^–1^, among the established click chemistry classes.^17^ CuAAC operates under mild conditions in aqueous environments^18^ and exhibits notable regioselectivity, backed by well-established mechanistic understanding.^19^ However, the use of copper catalysts in in vitro and in vivo experiments may introduce toxicity concerns, as even slight excesses of 10 mM in in vivo cellular concentrations within cells have the potential to cause neurologic and renal complications due to copper overload.^20^

**Table 1:**
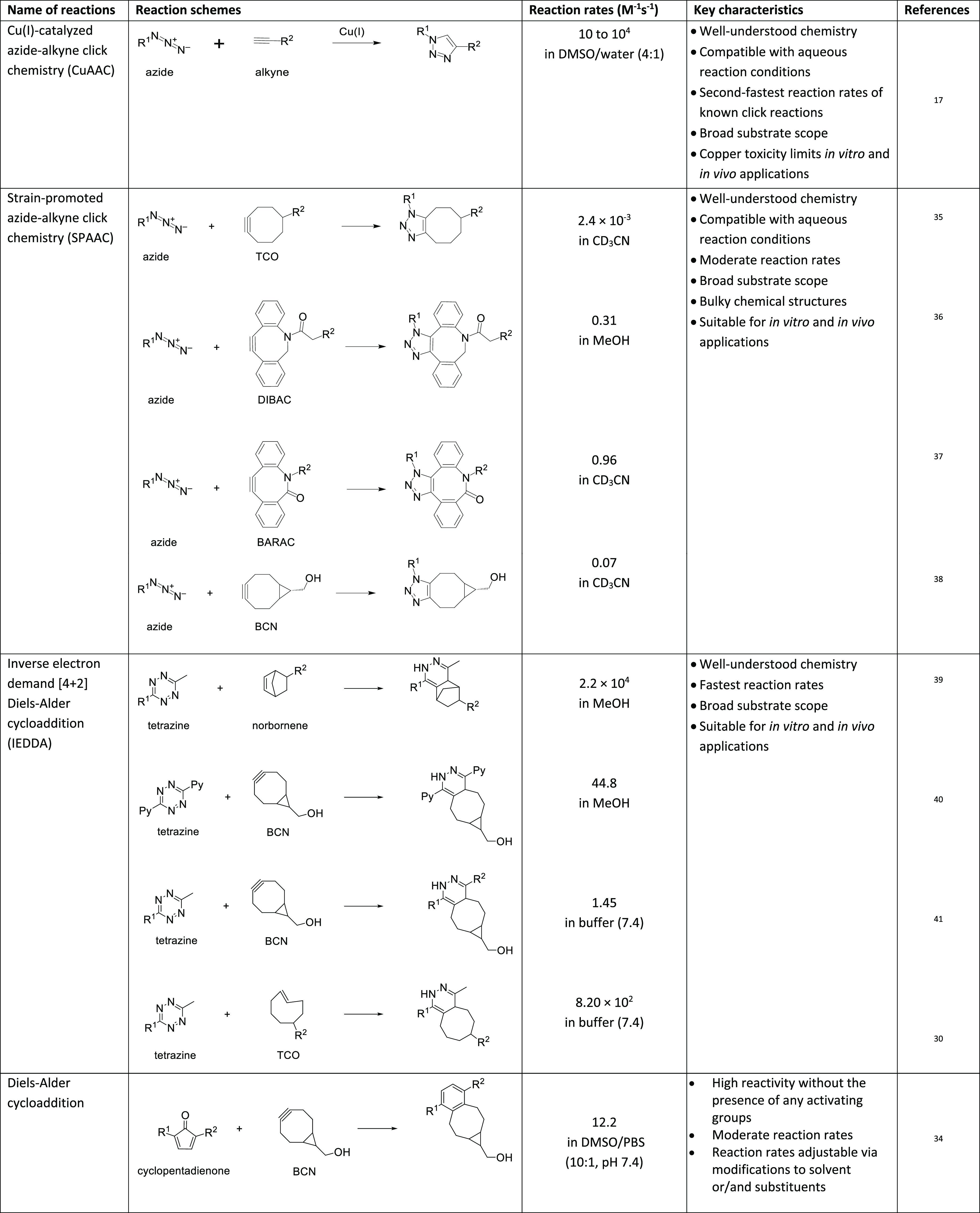
Overview of Well-Established Click Chemistry Reaction Classes, Including Key Characteristics and Reaction Rates^17,35,36,37,38,39,40,41,30,34^

The development of copper(I)-stabilizing ligands and incorporation of reducing agents has largely mitigated the concerns regarding the biotoxicity of copper(I). Reducing agents act to maintain a sufficient concentration of the catalytic copper(I) and prevent the formation of oxidative coupling products.^21^ Sodium ascorbate is often the preferred reducing agent over ascorbic acid, due to long-term stability issues associated with the air-oxidation of ascorbic acid, although it is an efficient reducing agent when oxygen exposure is minimized.^22^ Copper-stabilizing ligands act to enhance the efficiency, selectivity, and stability of CuAAC reactions through coordination of the copper(I), thereby preventing oxidation and undesired side reactions.^23^ The first generation of ligands, such as tris((1-benzyl-4-triazolyl)methyl)amine (TBTA), have limited water solubility, while newer generations, such as tris-hydroxypropyltriazolylmethylamine (THPTA) and 2-(4-((bis((1-(*tert*-butyl)-1*H*-1,2,3-triazol-4-yl)methyl)amino)methyl)-1*H*-1,2,3-triazol-1-yl)acetic acid (BTTAA), have significantly greater water solubility.^24^ The use of stabilizing ligands may increase the rate of the catalytic process, hence the ratio between the copper catalyst and stabilizing ligand needs to be optimized to balance stability and reactivity.^25^

Strain-promoted azide–alkyne cycloaddition click chemistry (SPAAC) reactions provide an alternative method to address the limitations of CuAAC reactions for in vivo applications by eliminating the need for a copper catalyst. SPAAC reactions employ strained-ring alkynes, which release high enthalpic energy with minimal input to form the [3+2] cycloaddition ring.^26^ Commonly used ring-strained alkynes include *trans*-cyclooctyne (TCO), dibenzoazacyclooctyne (DIBAC), biarylazacyclooctynone (BARAC), and bicyclo[6.1.0]non-4-yne (BCN) (Table 1, entry 2). Due to the absence of a copper catalyst, SPAAC reactions are more widely applicable in biological settings, particularly in in vivo labeling.^27^ However, SPAAC reactions proceed approximately 100-fold slower compared to CuAAC reactions.^28^ Additionally, their incorporation into biomolecules may present challenges due to their bulky structural attributes leading to steric hindrance, increased hydrophobicity, and impeding cellular penetration.

**Figure 2:**
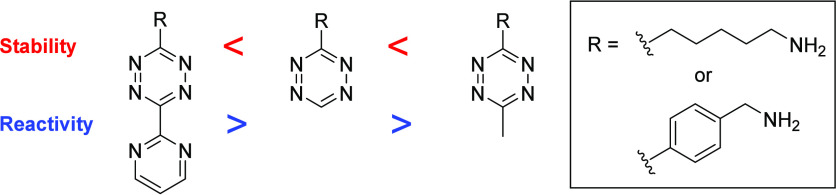
Stability and reactivity of selected tetrazines with TCO in PBS at 37 °C; R represents a benzyl amine or *n*-pentyl amine substituent. Data sourced from Karver et al.^30^

In response, the inverse electron demand [4+2] Diels–Alder (IEDDA) cycloaddition was developed. This ligation reaction involves electron-poor dienes, such as tetrazines and triazines, and electron-rich strained alkyne rings, like TCO, norbornene, and BCN (Table 1, entry 3). IEDDA reactions exhibit the fastest kinetics among known click chemistry reactions, with rate constants ranging from 1 to 10^6^ M^–1^ s^–1^ in water at 25 °C.^29^ The kinetics of IEDDA cycloadditions can be adjusted by modifying tetrazine ring functional groups, as less stable tetrazine compounds undergo IEDDA cycloadditions more rapidly (Figure 2).^30^ Additionally, dienes with higher electron deficiency yield greater reaction rates,^31^ while tetrazine rings with electron-withdrawing groups exhibit more than 20-fold higher reaction rates than tetrazines with electron-donating groups.

A newer version of the Diels–Alder cycloaddition is the strain-promoted azide–alkyne reaction of cyclopentadienones. Cyclopentadienones are considered to be one of the most reactive Diels–Alder dienes, differing from other Diels–Alder dienes due to the absence of electron-withdrawing heteroatoms.^32^ The kinetics of these reactions can be adjusted by varying solvent and functional groups, resulting in rate constants from 10^–3^ to 10^3^ M^–1^ s^–1^.^32^ Water has been shown to increase reaction rates due to hydrophobic aggregations and hydrogen bonding that is influenced by the increased polarization of the carbonyl moiety, resulting in up to 1000-fold higher reaction rates compared to reactions in isooctane.^32,33^ Similarly to IEDDA cycloadditions, strong electron-withdrawing groups on the cyclopentadienone, such as phenols and esters, afforded higher reaction rates, due to the electron-withdrawing groups decreasing the gap between the LUMO of the diene and the HOMO of the dienophile.^34^

The following section focuses on other well-studied click reaction classes, which are not frequently used in biological applications, including nucleophilic ring-opening reactions, non-aldol carbonyl reactions, and additions to carbon–carbon multiple bonds. Table 2 provides an overview of the reaction rates and key features of these click chemistry reactions. While less commonly used in biological experiments, it is important to understand the range of click chemistry methods available, and their benefits and limitations. These reactions typically exhibit lower reaction rates and/or selectivity. Nevertheless, these reactions remain useful for applications where rapid reaction rates do not limit their application.

**Table 2:**
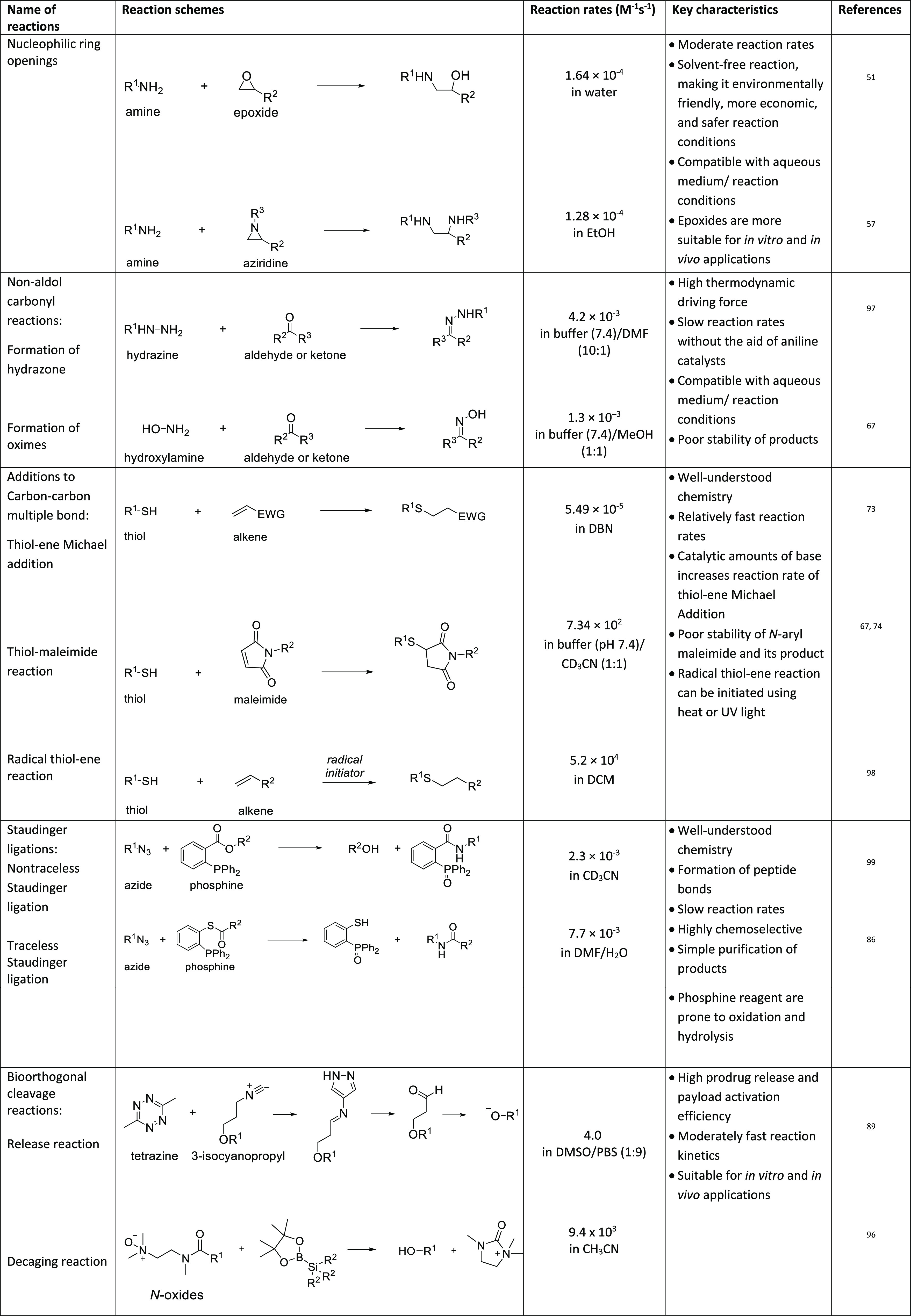
Overview of Less Conventional Click Chemistry Reaction Classes, Including Key Characteristics and Reaction Rates^51,57,97,67,73,74,98,99,86,89,96^

### Nucleophilic Ring-Opening Reactions

Nucleophilic ring-opening reactions involve breaking the strained electrophilic heterocycles such as epoxides, aziridines, cyclic sulfates, and episulfonium ions. Among these, epoxides and aziridines are the prevailing substrates for nucleophilic ring-opening reactions. Due to their inherent ring strain, they exhibit notable electrophilic reactivity, which can be further increased by introducing Lewis acids to coordinate with the oxygen atom.^42^ This interaction lowers the activation barrier for ring opening, resulting in an exothermic reaction.^43^ The process can be conducted without solvents or in an aqueous environment and demonstrates a stereoelectronically disfavored competing elimination process, avoiding the formation of side products. These characteristics facilitate high yields and simple product isolation, meeting the criteria for click reactions.^1,44^

Epoxide reactions are crucial in organic synthesis due to the high reactivity of the oxygen-containing three-membered ring to synthesize structural polymers.^45^ The reactivity stems from the polarity and angle strain, facilitating interactions with various Lewis acids and nucleophilic reactants, such as amines, alcohols, and thiols, to form β-amino alcohols,^46^ β-hydroxyl alcohols, and β-hydroxy sulfides, respectively.^47^ Among these, β-amino alcohols serve as vital building blocks for biologically active products including antihypertensive drugs.^48^ Consequently, nucleophilic ring-opening reactions of epoxides with amines (Table 2, entry 1) represent a crucial synthetic route, serving as the most practical and widely employed method for synthesizing β-amino alcohols. Their rate constants range from 10^–3^ to 1 M^–1^ s^–1^, with specific rates potentially influenced by both the solvent and nucleophilic reactants.^49^ These reactions are commonly conducted in an aqueous environment, which has shown to act as a catalyst, increasing the reaction rate, whereas dry organic solvents have shown limited success.^50^ However, water, acting as a nucleophile, may also react with epoxides, leading to hydrolysis and the addition of water molecules. To improve reaction efficiency, particularly in aqueous solutions, the pH can be increased above the p*K*_a_ of the specific amine or nucleophilic reactant, ensuring the complete conversion of the epoxide into the primary amine product without the formation of hydrolysis byproducts.^51^ Other factors influencing the rate constant include the strength of the acid, the ability of the acid to donate a hydrogen to the oxygen atom on the epoxide, and the presence of other nucleophiles (e.g., sulfates) which may assist in the reaction process.^49^

Aziridine reactions are also nucleophilic ring-opening reactions that form the foundation of many nitrogen-containing biologically active compounds such as antivirals (e.g., oseltamivir phosphate^52^) and antifungals (e.g., clotrimazole^53^). Similar to epoxides, aziridines are three-membered rings with elevated reactivity due to the incorporation of a nitrogen atom, altering their proton affinity. The reactivity of aziridines hinge on the substituent from the nitrogen, influencing the electrostatic potentials around both nitrogen and carbon atoms in the aziridine rings.^54^ Electron-withdrawing substituents facilitate ring-opening by releasing the electron density around the nitrogen, while electron-donating substituents do not readily react with nucleophiles. Lewis acids are employed to generate an aziridinium ion, enhancing the reactivity of aziridines with nucleophiles.^55^ Aziridine ring-opening reactions demonstrate reaction rates comparable to those of epoxide ring openings. Specifically, electron-donating substituents, such as *p*-methoxyphenyl (Table 2, entry 1), enhance the reaction rate 5-fold compared to electron-withdrawing groups such as *p*-chlorophenyl.^56,57^ Aziridine ring-opening reactions can be performed in a solvent-free environment and are not air- or water-sensitive, allowing the entire synthetic process to be carried out in a single vessel, bypassing the need for multiple steps, as changes in the reaction conditions have shown minimal effect on the overall aziridine ring-opening process.^58^

### Non-aldol Carbonyl Reactions

Non-aldol carbonyl reactions that form ureas, aromatic heterocycles, hydrazones, and oxime ethers use carbonyl functional groups to form aldol products without aldol condensation.^59^ Notably, formed compounds of hydrazones and oximes exhibit heightened stability and thermodynamic driving forces that result in reduced byproduct formation.^60,61^

Non-aldol carbonyl reactions involve aldehydes or ketones reacting with nucleophilic alkoxyamine or hydrazines, yielding oximes or hydrazones (Table 2, entry 2). These reactions are highly chemo-selective, producing only water as byproduct.^19^ Generally, aldehydes exhibit a higher reactivity compared to ketones, with a rate constant of 10^–3^ M^–1^ s^–1^.^20^ Reaction rate is dependent upon pH, as decreasing the pH to 3 increases the rate of reaction, although below pH 3, the rate begins to decelerate.^62^ Hydrazones and oximes are more hydrolytically stable than imines due to the adjacent heteroatom (N or O), thereby inducing an effect that reduces the nitrogen’s basicity. Limitations of non-aldol carbonyl reactions include product stability and slow reaction rates. Hydrazones are susceptible to dissociation at low concentrations,^63^ making oxime formation more favorable due to the increased stability the product.^63^ Recent studies in optimizing non-aldol carbonyl reactions suggest that the inclusion of anilines catalytically enhances yield efficiency and reaction rates up to 100-fold.^64^

Owing to their capability for in situ ligations within living organisms,^65^ hydrazines and oximes present opportunities for integration into bioconjugation applications. However, these reactions are less commonly utilized compared to other click reactions due to the challenges in synthesizing and storing the non-aldol clickable compounds. Specifically, aldehyde intermediates are susceptible to oxidation or self-reaction,^66^ further complicating their practical applications.

### Additions to Carbon–Carbon Multiple Bonds

Additions to carbon–carbon multiple bonds are nucleophilic addition reactions that activate alkenes and alkynes, exhibiting rate constants from 10^–2^ to 10^4^ M^–1^ s^–1^.^67^ Nucleophiles based on amines and thiols have shown substantial development in biomedical applications compared to other nucleophilic species, largely due to the prevalence of these compounds in naturally occurring structures (e.g., amino acid residues in proteins or biopolymers).^68,69^ Amine and thiol nucleophiles are extensively used in conjugation of biological compounds in medicinal chemistry applications.^70,71^

Michael additions involve nucleophilic addition to an α,β-unsaturated carbonyl compound containing an electron-withdrawing group (Table 2, entry 3). The electron-withdrawing group aids in stabilizing the intermediate carbanion, allowing for efficient nucleophilic addition. This approach is widely employed for forming carbon–carbon bonds due to chemo-selectivity, yielding stable products under mild conditions at room temperature.^67^ Thiols, known for their nucleophilicity, are commonly used in thiol–ene reactions as click Michael reactions, resulting in the formation of a thioether with the assistance of catalytic amounts of base.^69^ The reaction kinetics are influenced by factors such as the type of electron-withdrawing group on the carbon–carbon bond, base strength, solvent polarity, pH, and the thiol group orientation.^72^ For example, employing a stronger base like 1,5-diazabicyclo[4.3.0]non-5-ene (DBN) as opposed to triethylamine significantly increased the reaction rate by 3 orders of magnitude to 10^–3^ M^–1^ s^–1^ while requiring a lower concentration.^73^ Another commonly used thiol–ene reaction is the thiol–maleimide reaction, which exhibits relatively fast reaction kinetics up to 10^2^ M^–1^ s^–1^ and high selectivity in aqueous environments.^67^ Thiol–maleimide reactions are used for cross-linking of hydrogels and fluorescent labeling of molecules with the ability to undergo retro reactions at high temperatures to form succinimide thioether bonds.^72^ The poor stability of the formed products and the *N*-aryl maleimide reagent, which is more susceptible to hydrolysis compared to other thiol–ene reaction compounds, are some of the downsides of this reaction.^67,74^ However, solvents with higher polarity can promote the formation of thiolate ions, enabling the reaction to proceed in the absence of a catalyst, resulting in decreased reaction time and greater overall conversion.^75^

The radical-mediated thiol–Michael addition involves self-initiation through heat, ultraviolet (UV) light, or radical initiators to form thiyl radical species. Subsequently, these species propagate with the alkene group to form an intermediate carbanion, which then expels a hydrogen from a thiol and generates a new thiolate anion, allowing the cycle to repeat until all alkenes are consumed. This reaction is particularly effective in synthesizing polymer networks due to the ability to efficiently form a chain through repeated additions.^76^ Unlike other radical-mediated reactions, the thiol–ene radical reaction remains unaffected by oxygen, enabling it to occur under mild conditions and in common solvents, including water.^77^ However, careful consideration of reaction rate is required to minimize cytotoxicity^78^ and protein damage,^79^ especially during the generation of free-radical species.

### Staudinger Ligations

Staudinger ligations represent click reactions that have applications in protein and biosensor synthesis,^80^ due to their bioorthogonality, selectivity, and efficiency.^81^ The Staudinger ligation, derived from the classic Staudinger reaction,^82^ involves the interaction between an azide and an ester-derivatized phosphine, resulting in the formation of an iminophosphorane. This is rapidly followed by an intramolecular cyclization reaction, leading to the formation of an amide bond and the release of an alcohol byproduct (Table 2, entry 4).^83^ The two main types of Staudinger ligations are nontraceless and traceless variations.

In nontraceless Staudinger ligations, the desired amide bond is formed through intramolecular cyclization and spontaneous hydrolysis in aqueous media, incorporating the byproduct, phosphine oxide, into the structure.^84^ Based upon the same principles, the traceless Staudinger ligation presents a more refined form of this reaction, where the phosphine ligand is removed from the product after the ligation. The key difference, compared with the traceless reaction, is in the formation of the aza-ylide intermediate via the acylation of the phosphane group. This intermediate subsequently reacts with the azide, enabling the nucleophilic nitrogen atom of the aza-ylide intermediate to attack the carbonyl group and cleave the linkage with the phosphonium species.^85^ Hydrolysis leads to the dissociation of the phosphane oxide, which results in a phosphorus-free product. This reaction can occur under ambient conditions with high chemoselectivity.^85^ Staudinger ligations often exhibit slow reaction rates, with a rate constant of 10^–3^ M^–1^ s^–1^.^86^ The reaction rate can be increased by performing the reaction at an elevated temperature of 50 °C, but high temperatures are incompatible with most in vivo applications. Furthermore, phosphine reagents are prone to oxidation, leading to a competing side reaction that results in the elimination of the reactive phosphine species in the Staudinger ligation.

### Bond Cleavage Reactions

Novel click chemistry and bioorthogonal methodologies are constantly expanding the toolbox of available reactions. Bioorthogonal cleavage reactions are a noteworthy rapidly expanding approach.^87^ This reaction type allows for a controlled breakage where the function of the target molecule can be temporarily masked by a chemical cage that can be deprotected by a chemical decaging trigger restoring activity.^88^ While, still a relatively novel approach, significant progress has been made to improve the biocompatibility and efficiency of bond cleavage reactions, which exhibit moderately fast reaction rates, with a rate constant from 1 to 10^3^ M^–1^ s^–1^.

The use of 3-isocyanopropyl (Table 2, entry 5) and 3-isocyanopropyl-1-carbamonyl modifications as masking groups are examples for the controlled release of a biomolecule using bioorthogonal reactions.^89^ These groups can mask and cage a bioactive molecule, as well as release the cargo via a [4+1] cycloaddition when reacting with tetrazines, which releases nitrogen and forms a pyrazole-imine intermediate. Hydrolysis of the aldehyde facilitates the release of the biomolecule via β-elimination. The reaction products have shown to be stable under physiological conditions (i.e., PBS at 37 °C) and are nontoxic at concentrations as high as 100 μM. However, isocyanides undergo decomposition at room temperature, limiting their long-term storage.^90^

The click reaction between a tetrazine and *trans*-cyclooctene is the most successful click-to-release system, characterized by its fast reaction kinetics and highly efficient prodrug activation in both cells and mice.^91^ The first cancer treatment (SQ3370) utilizing click-to-release chemistry has successfully entered phase II clinical trials with promising phase I data on safety and localized activation by the human tumor sites.^92^ Building on this initial work, other research groups are trying to develop newer bioorthogonal reactions suitable for different in vivo applications.^93^*N*-Oxides have shown to have favorable biocompatibility properties and good stability under physiological conditions.^94^ Furthermore, *N*-oxides can be reduced in hypoxic tumor microenvironments, providing opportunities for targeted cargo release.^95^ Alternatively, boronic acids have been shown to effectively decage *N*-oxides under physiological conditions. To increase the reaction rate, Yan et al. recently reported a bioorthogonal decaging of *N*-oxide using silylboranes. The simple change from boronic acids to silylboranes resulted in a substantial 10^6^-fold acceleration in the reaction kinetics.^96^

## Reaction Kinetics for Applications of Click Chemistry

Click chemistry has been widely used across many areas of scientific research including biomedical imaging, medicinal chemistry, and nanomedicine, with applications of novel imaging probes for both in vitro and in vivo imaging, antibacterial, and anticancer agents. The adaptability of click chemistry has allowed the utilization of cycloadditions, nucleophilic ring-opening reactions, and Staudinger ligation reactions across a diverse range of applications, as evident from the plentiful library of research literature. For example, the click-activated drug delivery system by Shasqi has advanced from initial first human safety trials to phase 2 clinical trials, due for completion by the end of 2024.^100^ They have effectively labeled patients’ cells with a clickable handle and subsequently administered a drug or imaging agent linked to its complementary click functional group. This interaction ensures that the drug specifically targets the desired site. These encouraging outcomes suggest the potential of click chemistry for clinical applications.

One important but often neglected key factor in selecting an appropriate click reaction for an application is the reaction rate. This parameter determines the speed of product formation and reactant consumption while measuring the efficiency of a reaction. The progress of a reaction can be determined qualitatively or quantitatively. For example, visual changes such as reactant disappearance, color changes, and effervescence can be observed as qualitative analysis. Quantitative analysis includes determining the reaction rate of the decrease of the reactant concentration or the increase of the product concentration via the use of the rate equation. The rate equation has a rate constant, *k*, that describes the reaction speed. This constant varies based on reaction conditions including temperature, solvent, pH, nature of the reaction, and presence of catalysts. The rate of the reaction equation allows the rate-limiting reactant to be determined and the reaction order to be established.
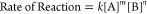
where [A] and [B] represent the reactants’ concentrations, and *m* and *n* represent the order of the reaction with respect to each reactant component.

Reaction rates can be divided into three categories: zero-, first-, and second-order reactions. In a zero-order reaction, elimination proceeds at a constant rate, independent of the reactant concentrations. First-order reactions depend on one reactant for elimination. Second-order kinetics relies on either the squared concentration of one reactant or the combined concentration of two reactants. Click reactions fall under the second-order reaction category, where their reaction rate is dependent on the activity of the two reactants.

Comprehending reaction rates guides the optimization of conditions to achieve efficient reactions, as it aids in predicting stability and reactivity of compounds under different applications. Consequently, emphasis on reaction kinetics enables informed decisions regarding reaction class, reactant concentrations, and reaction duration, increasing the likelihood of success in click chemistry applications. While there is a common desire for rapid kinetics, regiospecificity, easy purification, and simplicity in a reaction, it is important to acknowledge that not all applications require such rapid kinetics. Instead, the reaction kinetics must align with the relevant reactant concentrations as concentration can influence reaction time and efficiency. Consideration of reaction kinetics aids the selection of optimal conditions for each individual reaction.

Another important concept that is governed by its reaction kinetics is the idea behind enrichment-triggered prodrug activation. Wang et al. developed the idea of enrichment-triggered prodrug activation for controlled release of a biologically or clinically relevant agent.^101^ This work demonstrates that if two click partners are enriched in a specific target area, the local concentrations increase and enhance reaction rates, consequently resulting in the cargo release. However, the same two click partners do not undergo click reactions with each other or release the cargo while circulating at low concentrations. This demonstrated that reaction rates can be an important key in controlling the cargo/payload release, minimizing toxicity to nontargeted organs.

This review defines fast reaction rates as exceeding 30 M^–1^ s^–1^, resulting in nearly instantaneous completion. For instance, at initial concentrations of 10 μM for both reactants, 100 M^–1^ s^–1^ is calculated to have a half-life of approximately 0.28 h.^102^ Moderate reaction rates, ranging from 1 to 30 M^–1^ s^–1^, correspond to a half-life between 1 to 28 h, when using the same initial concentration of 10 μM.^102^ Slow kinetic reactions, characterized by values under 1 M^–1^ s^–1^,^27^ lead to reaction completion over extended time periods, ranging from days to years. For initial concentrations of 10 μM for both reactants, a rate of 1 M^–1^ s^–1^ yields a half-life of around 28 h.^102^

In this section of the review, detailed analysis of click chemistry applications, with respect to reported reaction rates, will be discussed for biomedical applications, including cancer therapy, pre-targeting approaches, and imaging probe implementation.

### Fast Reaction Kinetic Applications

To minimize interference with native biological processes in in vitro and in vivo environments, higher reaction rates might be advantageous to reduce impact on biological environment-sensitive molecules. Rapid kinetics are also favorable when the reaction involves chemically unstable compounds and when labeling applications require micromolar range concentrations.

**Figure 3:**
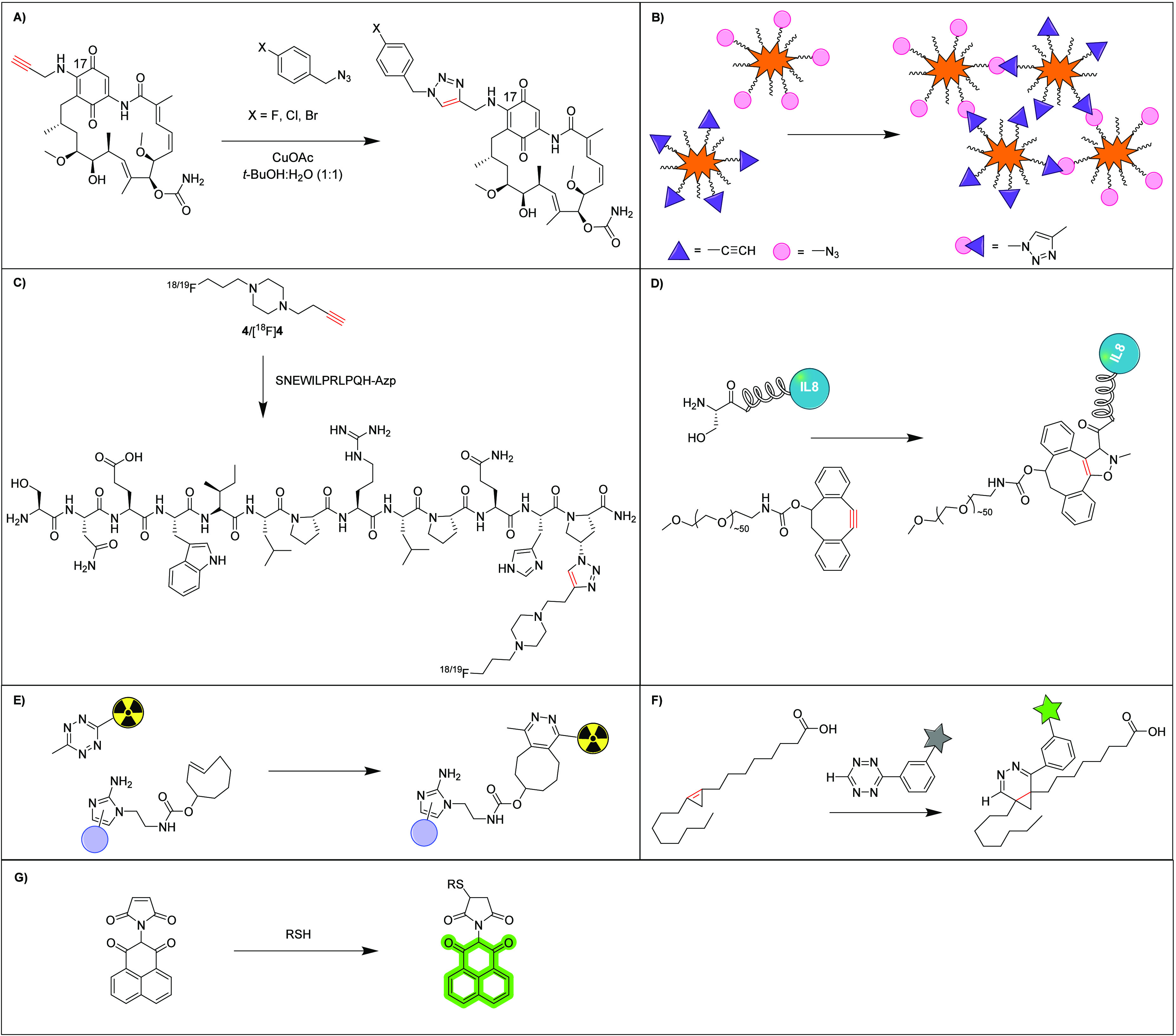
A) Reproduced scheme of CuAAC reaction between C(17)-alkyne and benzyl-halide.^103^ Copyright 2024 American Chemical Society. B) Reproduced schematic of DSPE-PEG-based azide- and alkyne-functionalized micelles undergoing CuAAC reaction with permission from ref (104). Copyright 2024 Elsevier. C) Reproduced scheme of the CuAAC reaction between [^18/19^F]BFP 4[^18^F]4 and SNEW peptide using DMF, sodium ascorbate CuSO_4_·5H_2_O, 40 °C, 16 h from ref (107). Copyright 2024 John Wiley and Sons. D) Reproduced scheme of one-pot SPANC reaction of IL-8 with PEG-cyclooctyne using: 1. NaIO_4_, NH_4_OAc buffer, pH 6.9, rt, 1 h; 2. *p*-MeOC_6_H_4_SH, rt, 2 h; 3. *p*-MeOC_6_H_4_NH_2_, MeNHOH·HCl, rt, 20 min; 4. rt, 20 h from ref (112). Copyright 2024 John Wiley and Sons. E) Reproduced scheme of reaction between radiolabeled tetrazine and TCO-bearing targeting molecule in hypoxic cells where blue spheres are the intracellular macromolecules.^113^ Copyright 2024 American Chemical Society. F) Reproduced scheme of sterculic acid reacting with tetrazine-fluorophore via IEDDA reaction where the star represents the fluorophore, BODIPY-FL, from ref (114). Copyright 2024 John Wiley and Sons. G) Reproduced scheme of the maleimide-1,8-naphathlimide reaction mechanism with thiol-active biomolecules.^116^ Copyright 2024 Elsevier.

CuAAC is widely considered the prototypic click reaction and remains a very active area of research due to its rapid kinetics and the utilization of small and synthetically easily accessible functional groups, such as azides and alkynes. Skrzypczak et al. recently reported the modification of the antineoplastic antibiotic geldanamycin through CuAAC, such that anticancer activity was increased across several cell lines while cytotoxicity to healthy cells remained unchanged.^103^ Following alkyne functionalization of the core of geldanamycin, CuAAC reactions were performed with an azide-tagged benzyl cap substituted with Cl, Br, or I (Figure 3A), creating geldanamycin-triazole-benzyl halogen derivatives. The IC_50_ values were determined to be approximately 0.2 (Cl), 0.3 (Br), and 0.4 (I) μM across SKBR3 (breast), SKOV3 (ovary), and PC3 (prostate) cancer cell lines, respectively, compared to approximately 0.5 μM for the unmodified geldanamycin.^103^ The aim of the study was to identify molecules with optimized lipophilicity and water solubility profiles, while modifying ligand interactions with target protein HSP90. In this case study, CuAAC provided fast access to a library of compounds with diverse substituents at the C(17)-position. Binding with the ATP-binding pocket of Hsp90 was improved with the novel tetrazine analogues, whereas other click chemistry reactions with bulkier reaction partners might have impeded binding to the target protein.

Other approaches to developing anticancer agents include the synthesis of nanoparticles for precision targeting of many tumor types. Research by Mei et al. highlighted the difficulty for typical intravenously administered therapeutic nanoparticles (approximately 100 nm) to enter the much finer lymphatic system, which is a key pathway involved in metastasis and therefore an important route to access tumor cells. The trade-off between a nanoparticle being small enough to penetrate malignant tissue but large enough to be well retained within the tumor inspired the development of a nanodrug delivery system with adjustable particle size. 1,2-Distearoyl-sn-glycero-3-phosphoethanolamine-polyethylene glycol (DSPE-PEG)-based micelles (25 nm) with modified azide- or alkyne-bearing surfaces were loaded with the chemotherapeutic agent, Paclitaxel (PTX). Following the addition of the catalysts copper(II) sulfate and sodium ascorbic acid, the micelles formed aggregates with one another (up to 120 nm) via CuAAC reactions (see Figure 3B). CuAAC was chosen due to the rapid kinetics that allowed the formation of the aggregates, increasing the tumor accumulation in the lymphatic system, whereas usually, nanoparticles are not the right size for drug delivery into the lymphatics. The in vitro analysis in murine breast cancer 4T1 cells verified that the smaller nonaggregated nanoparticles underwent exocytosis 1.74-times faster than the larger aggregates, resulting in greater retention of PTX following the CuAAC-mediated formation of nanoparticle aggregates. Further analysis in an in vivo 4T1 breast cancer mouse model revealed a 66.7% decrease in lymphatic metastasis, due to enhanced cytotoxicity resulting from greater PTX retention.^104^

CuAAC reactions have also shown promise in radiolabeling procedures for attaching radioisotopes to high-molecular-weight molecules (e.g., peptides). Radiolabeling presents unique challenges, such as the limited availability of radioisotopes and natural decay of radioactivity. Therefore, the kinetics of click bioconjugation must align with the decay rate of the radioisotope. Among the commonly employed radionuclides is the short-lived positron emitter fluorine-18 (^18^F), favored for its advantageous nuclear and chemical characteristics and applicability in positron emission tomography (PET) scans as almost all its energy can be utilized, minimizing dose requirements in patients.^105^ However, the incorporation of ^18^F into biomolecules presents challenges due to the harsh reaction conditions, i.e., high temperatures and basic environments, making it not ideal for biological settings.^106^ CuAAC reactions have emerged as valuable means for radiolabeling proteins with ^18^F under more moderate reaction conditions. However, the use of copper in these reactions may inadvertently lead to the formation of peptide complexes involving amino acid residues like serine and histidine, as observed by Pretze et al.^107^ The group developed a novel method to label ephrin (Eph) ligands, such as SNEW peptides, with radioisotopes by using CuAAC reactions. Adjusting the conditions to 40 °C for 16 h to conjugate ^18^F to peptides containing the amino acid sequence of SNEW effectively mitigated the formation of peptide complexes, affording products in high yields (see Figure 3C). The SNEW sequence acts as a high-affinity antagonist ligand for targeting ephrin type-B2 (EphB2) receptors, known to be overexpressed in various types of cancers (e.g., gastric, colorectal, and brain cancers). Imaging this biomarker by PET scanning could enable early tumor detection and monitoring responses to therapies directed toward EphB2 receptors to improve patient progress.

An important consideration for scaling bioconjugation is the increasing amount of copper(I) catalysts that is required to accelerate the reaction. Toxicity concerns may arise for in vitro applications where click products contain copper(I) impurities, and in vivo applications where copper radicals degrade peptides and proteins during the click reaction,^108^ limiting the use of copper catalysts in live cells or organisms. To overcome these limitations for many in vivo applications, alternative rapid click reactions, such as strained-promoted alkyne–nitrone cycloadditions (SPANC), have emerged with comparable rate constants, reaching up to 47 M^–1^ s^–1^.^109^ Starting materials of SPANC reactions are more stable, as they are not susceptible to hydrolysis. SPANC reactions are primarily employed in functionalizing the N-terminus of proteins that contain a serine residue for conjugation to the protein of interest.^110^ This method proves effective in labeling the N-terminus due to the fast reaction rate, avoiding denaturation of the protein, while modifying only a single site. Unlike conventional approaches for targeting cysteine and lysine residues, which leads to dimerization and loss of function, SPANC provides a more selective alternative.^111^ For example, SPANC was used to modify the chemokine interleukin-8 (IL-8) with a nitrone group prior to a click reaction with cyclooctyne. Nitrones containing ester or amide substituents exhibited faster kinetics, reaching up to 39 M^–1^ s^–1^ (Figure 3D).^112^ This reaction provides an alternative labeling approach for incorporating radioisotopes for imaging IL-8.

The utilization of IEDDA chemistry for pre-targeting approaches has gained great traction in recent years, with varying degrees of success. Pre-targeting allows a bioorthogonal-handled targeting vector (e.g., antibody) to be administered and reach optimal biodistribution prior to the introduction of a complementarily tagged cytotoxic cargo (e.g., drug, radioisotope), thereby reducing negative impacts such as off-target drug effects and dosimetry concerns resulting from high activities of radioisotopes. A recent study by Allot et al. demonstrated the utilization of TCO-tetrazine (Tz) IEDDA chemistry to selectively increase ^18^F radiotracer uptake in hypoxic (0.1% CO_2_) versus normoxic (5% CO_2_) live cells in a pretargeted approach (Figure 3E). The small molecule targeting vector 2-nitroimidazole, which is known to accumulate preferentially in hypoxic cells, was TCO-modified and incubated with EMT6 and HCT116 cancer cells for 12 h. Next, cell-membrane-permeable [^18^F]FB-Tz was administered and incubated for a further hour to allow retention of the radiotracer in the TCO moiety-containing cells and subsequent IEDDA reaction. Gamma counting of the both cell lines treated with a 1–10 μM concentration of TCO-2-nitroimidazole revealed a significantly greater amount of ^18^F in the hypoxic (approximately 90% of ID/mg) compared to the normoxic (<10% ID/mg) cells. Furthermore, direct comparison of cellular uptake of [^18^F]FB-Tz compared to the “gold standard” cellular hypoxia-detecting radiotracer, [^18^F]FMISO, revealed that, while the absolute uptake of [^18^F]FB-Tz was significantly greater in both cell lines, the hypoxic:normoxic uptake ratio was higher for [^18^F]FMISO. It was suggested that this may be due to the lipophilic nature of [^18^F]FB-Tz causing nonspecific uptake.^113^

IEDDA chemistry also facilitates bioconjugation reactions on a second-to-minute time scale, reducing the required concentration of reagents for live cell labeling. In the field of lipid research, these attributes make IEDDA reactions highly advantageous for investigating unsaturated free fatty acids in living cells. The study of lipids presents many challenges owing to their lipophilic nature, and chemical modifications that lead to substantial alterations in their structural and biochemical properties.^106^ Thus, the application of click chemistry to lipid studies allowed for minimal structural adjustments. Bertheussen et al. employed IEDDA reaction involving tetrazines and cyclopropenes for lipids analysis (Figure 3F).^114^ Cyclopropenes were favored over TCO due to their smaller size, reducing the likelihood of structural modification while maintaining steric hindrance. This approach exhibited fast ligation kinetics of 660 M^–1^ s^–1^, enabling imaging in both live and fixed cells, facilitated by the tetrazine ring’s quenching effect on fluorophores. In live cells, dendritic cells were labeled with cyclopropenes, followed by IEDDA reactions with the tetrazine-fluorophore, allowing unsaturated lipids to undergo proteomic analysis and be visualized using live-cell microscopy. This work demonstrated the feasibility of employing this technique in live cells for investigating fatty acid uptake by using click reactions and fluorescence.

Finally, thiol–maleimide reactions have shown success in tracking thiol-containing molecules, including cysteine, homocysteine, and glutathione.^115^ For example, Qu et al. have synthesized a maleimide-containing 1,8-naphthalimide fluorogenic probe (Figure 3G) to target thiols in cells.^116^ Increase in fluorescence intensity was used to track the reaction with thiols. Additional results showed that the thiol–maleimide reactions are highly selective toward cysteine, homocysteine, and glutathione but not toward other natural amino acids or metal ions. Experiments in HepG2 cells showed good membrane permeability of the probe and its ability to label thiols within living cells. Under the reported experimental conditions, a rapid kinetic response was observed, resulting in reaction completion with cysteine, homocysteine, and glutathione within 20 s. The fast reaction kinetics permitted visualization in real-time using bioimaging methods. Furthermore, the high reaction rates provide opportunities for quantitative detection without the need for pretreatment with samples.

### Moderate Kinetic Applications

Moderate kinetic reaction rates are considered when prioritizing stability of the reactants in solution and the environment in which the reaction will take place, e.g., in vitro or in vivo. Examples of applications that would require moderate reaction rates include the use of click chemistry in nanomaterials, anticancer agents and protein labeling in live cells.

**Figure 4:**
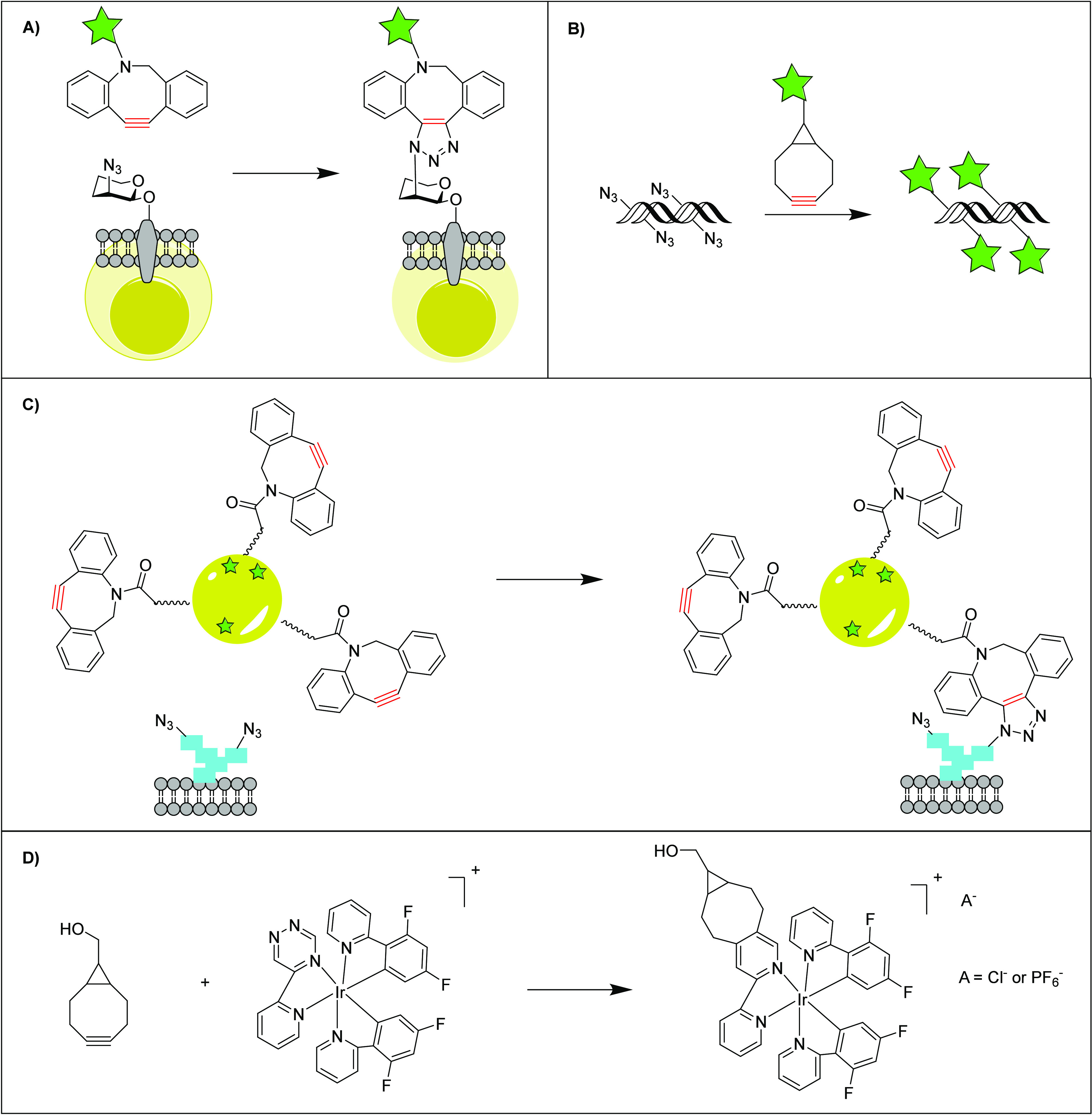
A) Reproduced scheme of azido group and near-infrared fluorescence (NIRF)-dye-labeled DIBAC where the yellow cell represents chondrocytes and the star represents the DIBAC-650.^118^ Copyright 2024 American Chemical Society. B) Reproduced scheme of double-stranded DNA linked with long azidoalkyl linker reacting with BCN-Cy5 (green star) via SPAAC reaction.^119^ Copyright 2024 Oxford University Press. C) Reproduced scheme of unnatural glycans modified with azides reacting with nanoparticles coated with DIBAC and Cy5 (green star) via SPAAC reaction where blue rectangles represent unnatural glycans with permission from ref (120). Copyright 2024 John Wiley and Sons. D) Reproduced reaction scheme of iridium(III) complexes 8Cl and 8PF_6_ with BCN.^121^ Copyright 2024 Royal Society of Chemistry.

Glycoengineering employs moderate reaction rates for modifying living cells with substrates for click chemistry in both in vitro and in vivo applications. This method is crucial for long-term cell monitoring in in vivo settings. Due to its moderate reaction rates, the SPAAC reaction is more stable, enabling effective tracking for up to 4 weeks at a rate of 1.2 × 10^–3^ M^–1^ s^–1^.^117^ For example, Yoon et al. performed a SPAAC reaction between azide-labeled chondrocytes and near-infrared (NIR)-dye-labeled dibenzyl cyclooctyne (DIBAC-650) (Figure 4A). Imaging revealed a 19-fold increase in fluorescence compared to the standard lipophilic fluorescent dye, DiD, over 4 weeks with lower cytotoxicity.^118^ The ability to locate and track chondrocytes over a period of 4 weeks suggests that click chemistry may be a viable method to monitor transplanted chondrocytes in bioengineered cartilage. Although cell imaging hindered the cartilage formation in vivo due to the potential disturbance of cell function and formation of cartilaginous tissues, the use of click chemistry helped reduce side effects on cartilage formation, showing promise for therapeutic tissue engineering applications.

SPAAC reactions have also shown promise in the imaging and detection of specific deoxyribonucleic acid (DNA) sequences. The small size of the azide groups, copper-free compatibility, and orthogonality to DNA functional groups are advantages of SPAAC; however, the bulky alkyne is suboptimal for DNA polymerases. To address this, a new approach was developed to synthesize fluorescently labeled DNA combining polymerase-based methods and SPAAC reaction. SPAAC reactions offer stability during postlabeling, unlike other click reaction types. Ren et al. demonstrated the effectiveness of extending the azidoalkyl linker on the DNA chain using polymerase enzymes.^119^ The extended linkers minimized and eliminated steric hindrance from the strained alkyne ring, BCN, and the fluorophore, Cy5, to the DNA chain (Figure 4B). It was found that the number of azidoalkyl linkers on the DNA chain affected the fluorescence intensity due to a fluorescence quenching mechanism. These observations emphasized the importance of the fluorescence labeling density on DNA chains. Utilizing SPAAC chemistry enabled control over the labeling density and minimized fluorescence quenching. Furthermore, the ability to dual-fluorescent label DNA strands allowed for simultaneous detection of multiple gene sequences by varying the ratio of fluorophores in the mixture. This method holds promise for advancing visualization of multiple gene sequences concurrently.

SPAAC reactions also have applications in nanoparticle formation. Koo et al. employed SPAAC chemistry for precise delivery of nanoparticles to tumors by utilizing glycoengineering techniques to introduce bioorthogonal chemical agents on cell surfaces.^120^ This, along with the prolonged nanoparticle circulation time of 3 days and slower reaction rate, increased nanoparticles binding to target cells in vivo. Consequently, exogeneous glycans were modified with azides, while nanoparticles were coated with DIBAC, enabling the SPAAC reaction. Upon intravenously administering the nanoparticles to lung-tumor-bearing mice, selective affinity for the azide group was observed, which was tracked via Cy5 fluorescence (Figure 4C). Through glycoengineering, the number of azides on the surface and, therefore, the shape and size of nanoparticles can be controlled in a dose-dependent manner. Remarkably, owing to the inherent glycan internalization, the nanoparticles could be internalized into cells following binding, demonstrating their potential use for intracellular drug delivery.

IEDDA reactions demonstrate the most rapid kinetics of all click reactions. While, 1,2,4,5-tetrazines are usually chosen due to their fast-kinetic rates, their stability is suboptimal under physiological conditions. Hence, recently, Kozhevnikov et al. investigated the slower IEDDA with 1,2,4-triazines, which showed excellent stability under physiological conditions. To accelerate the IEDDA with 1,2,4-triazine, Kozhevnikov et al. developed a novel 1,2,4-triazine-iridium(III) complex, with reaction rates greater than previously reported BCN kinetics. The triazine-containing ligands were prepared by coordinating iridium(III) with 5-(2-pyridyl)-1,2,4-triazine, and either two chloride or hexafluorophosphate-containing non-triazine units to complete the coordination (Figure 4D). The resulting ligands, termed 8Cl and 8PF_6_ respectively, were subsequently reacted with BCN to produce luminescent click products, with second-order rate constants of approximately 8 M^–1^ s^–1^. In contrast, the second-order rate constant of the uncoordinated 5-(pyridine-2-yl)-1,2,4-triazine ligand was determined to be several orders of magnitude lower at approximately 0.06 M^–1^ s^–1^.^121^ Thus, this example emphasizes the importance of balancing both kinetic and stability factors. While preliminary cell-based studies identified potential toxicity associated with the triazine ligand, the development of clickable anticancer agents might exploit these effects.

### Slow Kinetic Applications

Slow rate click reactions are normally composed of Staudinger ligations and the formation of either hydrazones or oximes. These reactions are suitable for applications where the coordination of the click reaction and relevant interactions have consecutive steps that need to be performed in a timely manner in order for the overall application to proceed. Slow kinetic reactions may also be utilized for initial proof-of-concept studies to determine the feasibility of click chemistry for in vitro and in vivo environments or a strategy to overcome bioavailability limitations of the more rapid click reactions CuAAC and IEDDA, rendering them unusable for in vitro and in vivo environments.

**Figure 5:**
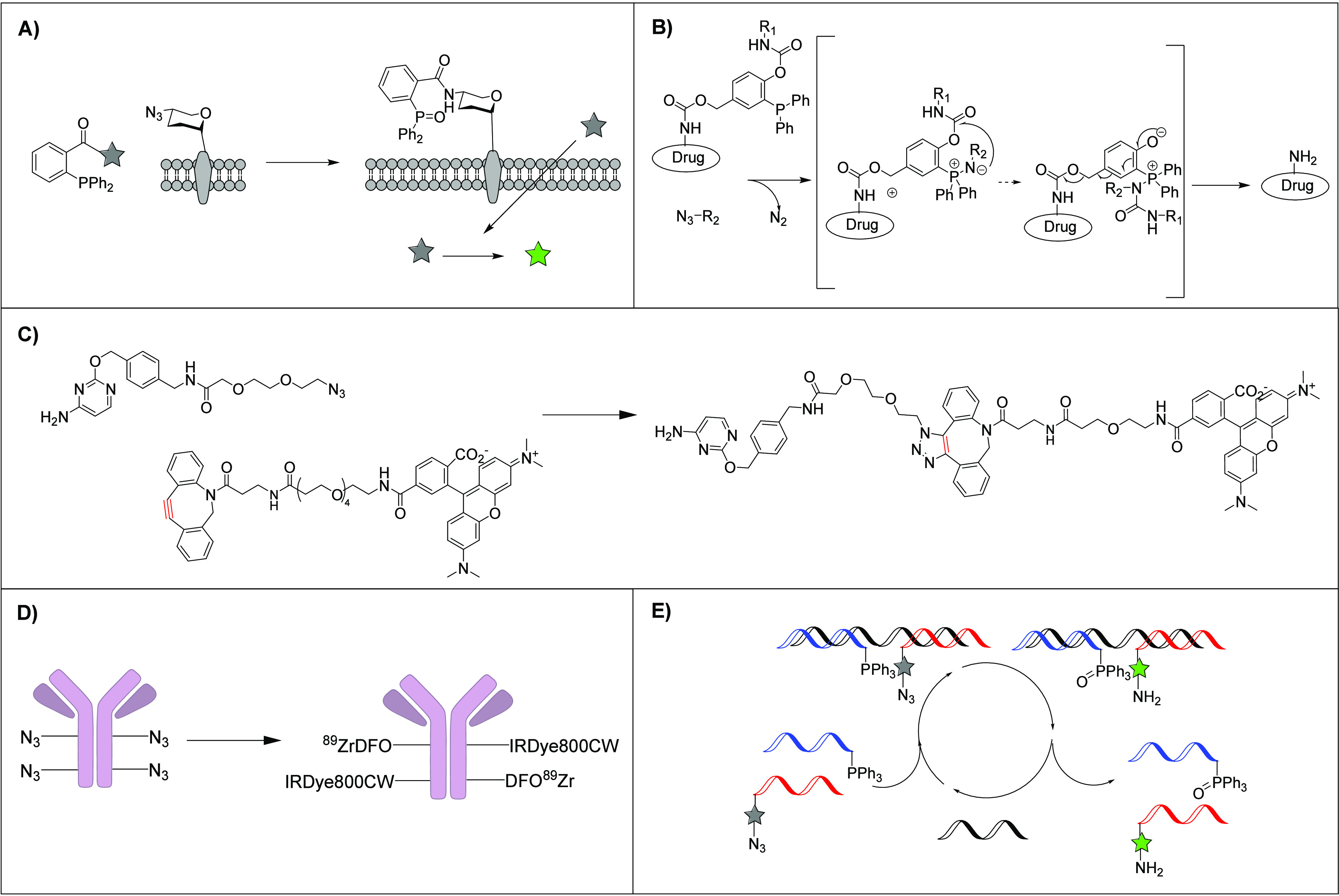
A) Reproduced scheme of the reaction between cell-surface azido-sugar with a compound via Staudinger ligation that releases luciferin (green star), which diffuses into the cell, where it is converted to bioluminescent oxyluciferin.^99^ Copyright 2024 American Chemical Society. B) Reproduced scheme of the mechanism of prodrug activation via Staudinger ligation where R_1_ is nitrobenzene and R_2_ is PEG3-amine from ref (124). Copyright 2024 Elsevier. C) Reproduced reaction scheme of BC-Azide (CLIP-Tag Substrate) and DIBAC-PEG_4_-6-TAMRA (fluorescent reporter).^125^ Copyright 2024 American Chemical Society. D) Reproduced reaction scheme of azide-modified anti-MT1-MMP antibody with DIBAC-tagged IRdye800CW and DFO.^126^ Copyright 2024 American Chemical Society. E) Reproduced scheme of the catalytic cycle of templated Staudinger reaction yielding a fluorescent product, where the black strand represents the temple, the blue strand represents the triphenylphosphine-PNA conjugate, and the red strand represents the masked-7-azidocoumarin-PNA conjugate from ref (127). Copyright 2024 The Royal Society of Chemistry.

Slow click reactions are suitable in applications in which reaction kinetics are not a primary concern. Specifically, Staudinger ligations outperform other click reactions for targeting azido sugars in mice despite their slow reaction kinetics, highlighting their significance in biomedical applications.^99^ This is important, as glycans serve as dynamic indicators of cell physiology.^122^ The evolution of the structure over time allows the observation of transitions from a healthy to a malignant state. Glycans can be monitored through modification of their structure with a compatible click reagent, which complexes with an imaging probe possessing a complementary click substrate. Evaluation of click reactions in mice found that those with rapid kinetics exhibited limited bioavailability and were less efficient in the model.^123^ Consequently, Staudinger ligations remain as a primary click reaction for glycan studies in mice. Notably, Cohen et al. used Staudinger ligations to monitor cell-surface azido sugars by releasing luciferin, a commonly used substrate for bioluminescence real-time animal imaging.^99^ This approach demonstrated its effectiveness, as Staudinger ligations successfully released luciferin, resulting in higher bioluminescence in cells with azido sugars compared with those without (Figure 5A). The second-order rate constant was determined to be 2.3 × 10^–3^ M^–1^ s^–1^, which equates to 2 h to achieve almost full conversions of azides. For in vivo cell-surface labeling, this is sufficiently stable for glycan applications. The signal intensity correlated directly with the concentration of azido sugar and probe, allowing for precise quantification and elimination of background signals. Furthermore, bioluminescence enabled real-time quantification of the system. This technique holds potential for in vivo imaging of azide-labeled azido sugars, offering a new platform for real-time monitoring and imaging of intracellular processes.

Azoulay et al. employed Staudinger ligation in the initial phase of their study to achieve controlled release of the active drug from a prodrug. They introduced modifications to the Staudinger ligation, enabling intramolecular cyclization and initiating a 1,6-elimination process to liberate the drug, doxorubicin, in its original state (Figure 5B).^124^ Their preliminary experimentation involved a model masked compound that demonstrated the release of the desired compound through the proposed mechanism upon reaction with the azido compound. Similarly, the prodrug doxorubicin exhibited enhanced doxorubicin levels within 30 min under the same system with the azido compound. This reached 90% completion after 3 h at 37 °C, indicating optimal in situ conditions. This study demonstrated the effective utilization of Staudinger ligation for the selective release of the drug in high yields despite slow kinetics.

SPAAC reactions may not be suitable for therapeutic pre-targeting approaches due to their relatively long reaction times, but they have been shown to be a useful tool in creating novel imaging probes. Macias-Contreras et al. demonstrated how incorporating a combination of SPAAC and IEDDA click chemistry can facilitate dual orthogonal/bioorthogonal intracellular labeling. Two proteins of interest, a nuclear envelope protein and a cytoskeletal protein, were first genetically modified using CLIP and SNAP self-labeling techniques. Synthetic nucleic acid compounds, benzylguanine (BG) and benzylcytosine (BC), that orthogonally bind SNAP and CLIP, respectively, were modified to bear SPAAC handles (DIBAC, cylopropene) and IEDDA handles (TCO, Tz, norbornene) (Figure 5C). Fluorophores bearing the bioorthogonal click handles were synthesized and administered to SNAP/CLIP modified cells. Fluorescence microscopy revealed selective labeling of the target proteins using several combinations of click chemistry handles bearing red and green fluorophores.^125^ These findings demonstrate a simple method for dual labeling of intracellular targets, as well as providing potential to expand the nature of the labeling cargo to include other compounds such as radiotracers.

SPAAC reactions have also proven successful in imaging radiotracers in an in vivo setting, as a recent study by Pringle et al. reported the development of a dual modality imaging probe targeting osteosarcoma, for both pre- and intraoperative imaging, as well as fluorescence-guided surgery. Here, an anti-MT1-MMP antibody underwent glycan modification to create four azide-functionalized sites, prior to conjugation of DIBAC-tagged IRDye800CW, and the zirconium-89 (^89^Zr) chelator, deferoxamine (DFO), via a SPAAC reaction in a site selective manner (Figure 5D). This bioconjugate demonstrated high in vivo stability in a novel osteosarcoma mouse model, largely due to the robust labeling of the antibody via SPAAC.^126^

Staudinger ligation has also been observed to exhibit suitable slow kinetics for oligonucleotide-templated probes. These probes guide the hybridization of two unreactive fragments through close-proximity-based pairing reactions. This reaction relies on the presence of the probes. While click reactions offer advantages for this type of reaction, it is important they exhibit low background noise and do not occur in the absence of the oligonucleotide template.^102^ Hence, Staudinger ligation possesses the appropriate reaction kinetics that allows the target-template to hybridize prior to the click reaction, unlike click reactions with rapid kinetics that may react in the absence of the oligonucleotide template when unreacted fragments are coincubated. Pianowski et al. employed this technique to catalytically and fluorescently detect the spatial proximity of oligonucleotide-templated probes.^127^ By reuse of the template, the conjugation of the two strands could be continued. The masked-7-azidocoumarin-peptide nucleic acid (PNA) conjugate became fluorescent after undergoing a Staudinger reaction with the triphenylphosphine–PNA conjugate (Figure 5E). This experiment confirmed that the template played a key role in enabling the click reaction, resulting in high fluorescence emissions compared to the absence of emissions when the two strands were coincubated without the template. This demonstrated the potential of employing additional Staudinger reactions in DNA-templated reactions, which warrants further investigation in the diagnostics of single base pair mismatch strands.

## Conclusion

The discovery of click chemistry and bioorthogonal chemistry has profoundly impacted modern chemistry. It has enabled efficient synthesis of nontoxic products suitable for diverse biology and drug discovery applications. While the majority of reviews in this field focus on the benefits of click reactions with high reaction rates, this review emphasizes that rapid reaction rates are not always a necessity for biomedical applications. Rather, click reactions should be fine-tuned to the specific applications and desired outcome. For example, slower click reactions allow the formation of more stable products, which is beneficial for creating imaging probes. Rapid reaction rates are more crucial for sensitive biological and chemical applications, especially when involving unstable biomolecules and sensitive biological processes. Given the ever-growing range of click-handled compounds that are commercially available or synthetically accessible, researchers can be more selective with their choice of click reaction handles. Ultimately, this review will aid those who are interested in the use of bioorthogonal click chemistry by giving a brief overview of the currently employed click reactions. By understanding the benefits and limitations of each type of click reaction, researchers can avoid pitfalls in their experimental and molecule design. The field of click chemistry is continuously growing and expanding the diversity of reactions with different reaction rates, which will be key to the development of new biomedical applications.

## Abbreviations

^18^F: fluorine-18
^89^Zr: zirconium-89
BARAC: biarylazacyclooctynone
BC: benzylcytosine
BCN: bicyclo[6.1.0]non-4-yne
BG: benzylguanine
BTTAA: 2-(4-((bis((1-(*tert*-butyl)-1*H*-1,2,3-triazol-4-yl)methyl)amino)methyl)-1*H*-1,2,3-triazol-1-yl)acetic acid
CuAAC: copper(I)-catalyzed azide–alkyne click chemistry
DBCO: dibenzylcyclootyne
DBN: 1,5-diazabicyclo[4.3.0]non-5-ene
DIBAC: dibenzoazacyclooctyne
DCM: dichloromethane
DFO: deferoxamine
DMF: *N,N*-dimethylformamide
DMSO: dimethylsulfoxide
DNA: deoxyribonucleic acid
DSPE-PEG: 1,2-distearoyl-sn-glycero-3-phosphoethanolamine-polyethylene glycol
Eph: ephrin
EphB2: ephrin type-B2
EtOH: ethanol
h: hours
IC_50_: half maximal inhibitory concentration
IEDDA: inverse electron demand [4+2] Diels–Alder
IL-8: chemokine interleukin-8
MeOH: methanol
min: minutes
NIRF: near-infrared fluorescent
PBS: phosphate buffer sulfate
PEG: poly(ethylene glycol)
PET: positron emission tomography
p*K*_a_: acid dissociation constant
PNA: peptide nucleic acid
PTX: paclitaxel
Py: 2-pyridine
s: seconds
SPAAC: strain-promoted azide–alkyne cycloadditions
SPANC: strained-promoted alkyne-nitrone cycloadditions
TBTA: tris((1-benzyl-4-triazolyl)methyl)amine
TCO: *trans*-cyclooctyne
THPTA: tris-hydroxypropyltriazolylmethylamine
Tz: tetrazine
UV: ultraviolet
